# Antiinflammatory, Analgesic and Antipyretic Activity of Certain Thiazolidinones

**DOI:** 10.4103/0250-474X.41448

**Published:** 2008

**Authors:** A. D. Taranalli, A. R. Bhat, S. Srinivas, E. Saravanan

**Affiliations:** Department of Pharmacology, K. L. E. S's College of Pharmacy, Belgaum-590 010, India; 1 Department of Pharmaceutical Chemistry, K. L. E. S's College of Pharmacy, Belgaum-590 010, India

**Keywords:** Thiazolidinones, antiinflammatory, analgesic, antipyretic, cyclooxygenase

## Abstract

The thiazolidin-4-one derivatives and the corresponding spiro compounds were synthesized from sulphanilamide and were evaluated for anti-inflammatory and analgesic activity in acute and sub acute models. Compounds were also evaluated for antipyretic and cyclooxygenase enzyme inhibitory activity. All the compounds showed significant antiinflammatory, analgesic and antipyretic activity at 100 mg/kg in all the models. The compounds B1, B2, B5, B6, and B8 showed maximum inhibition of COX-2 activity without inhibiting the COX-1 activity. The nimesulide was used as standard drug for comparison. The substitution at R, R_1_ and R_2_ with the functional groups Cl, OCH_3_, NO_2_ and OH in the aromatic ring resulted in increased activity as compared to unsubstituted thiazolidin-4-ones. However the substitution at R_3_ with spiro group did not improve the activity. The study suggests that COX-2 binding site may not be a rigid structure but might adopt to various related molecules.

Inflammation is defined as a tissue directed response to noxious and injurious external and internal stimuli, which is predominantly mediated by arachidonic acid metabolites. In the early 1990s two groups independently detected the existence of two cyclooxygenases COX-1 and COX-2^1^. While the inhibition of COX-2 activity led to antiinflammatory activity, COX-1 inhibition led to ulcerogenic activity. Non steroidal antiinflammatory drugs are a non homogeneous family of pharmacologically active compounds used in the treatment of acute and chronic inflammation, pain and fever. Review of experimental data reveals that subtle changes in the binding site of COX-2 might occur to adopt its structure to the inhibitor^2^,^3^. This might be the reason for many diverse group of compounds reported to have antiinflammatory activity^4^–^7^. The survey of newer CoX-2 inhibitors shows that the presence of sulphanamide and biphenyl groups which might be essential for COX-2 activity. Substituted thiazolidin-4-one derivatives have shown promising cyclooxygenase and 5-lipoxygenase inhibiting properties and used as topical antiinflammatory agents for inflamed conditions of skin. Moreover, the inflammation is a general condition associated with infection and sulphonamide group appears to be essential for antiinflammatory activity. Hence various derivatives of thiazolidin-4-ones synthesized were evaluated for their antiinflammatory, analgesic, antipyretic and cyclooxygenase inhibitory activities.

## MATERIALS AND METHODS

Wistar rats of either sex weighing 150-200 g were used. Animals were housed in groups of six per cage at a temperature of 25±1° and relative humidity of 45±5%. A 12:12 hour light:dark cycle was followed during the experiments. Animals had free access to food and water, however, food was withdrawn six hours before and during the experiments. The animals were obtained from the Central Animal House of J. N. Medical College, Belgaum (India). The Institutional Animal Ethical Committee approved the protocol of the study.

The drugs synthesized in the Chemistry laboratory of K. L. E. S's College of pharmacy, Belgaum, were used. The drugs were coded as B_1_ to B_8_ (thiazolidine-4-one compounds) and B_9_ to B_11_ (Spiro derivatives of thiazolodine-4-one) Table 1. The standard drug nimesulide was obtained from Lincoln pharmaceuticals Ltd. Ahamedabad.

**TABLE 1:** SHOWING VARIOUS SUBSTITUTIONS IN THIZOLIDINONE MOIETY

Compound code	R (R)	R’ (R1)	R” (R2)
B_1_	H	H	H
B_2_	H	OCH_3_	H
B_3_	CH_3_	Cl	H
B_4_	CH_3_	OCH_3_	H
B_5_	H	OCH_3_	OH
B_6_	H	Cl	H
B_7_	CH_3_	NO2	H
B_8_	H	F	H
B_9_	H	H	H
B_10_	H	OCH_3_	H
B_11_	CH_3_	OCH_3_	H

### Toxicity studies:

The acute toxicity study was done as per the OECD guidelines (407). The compounds were administered orally in different doses, where 24 h toxicity was recorded to identify the toxic doses. The doses of the test compounds were then fixed on the basis of their acute toxicity as 50 mg/kg and 100 mg/kg for evaluation. The antiinflammatory activity was studied using acute and chronic models.

### Carrageenan-induced paw edema^8^:

All the test compounds namely B_1_ to B_11_ were administered in two doses 50 mg/kg and 100 mg/kg body weight based upon their acute toxicity studies and nimesulide 50 mg/kg b.w. was used as standard. The test compounds were administered orally to the rats suspended in 0.5% carboxymethyl cellulose (CMC). The control animals received 0.5% CMC. Thirty minutes after drug administration, 0.1ml of 1% carrageenan (Sigma) in normal saline solution was injected into the subplantar region of one of the hind paws. The paw edema volume was recorded using a plethysmometer (UGO Basile, Italy) at different time intervals.

### Xylol-induced mouse ear edema^9^:

The test compounds, standard and vehicle as mentioned above were administered orally to the mice. Thirty minutes after administration, inflammation was induced by a topical application of 2% xylol (20μl) to the right ear of each mouse. The left ear was kept as control. The positive control group received only 0.5 ml of 1% CMC. After 30 minutes of xylol application, the animals were killed by cervical dislocation. A 6 mm section of ear disc was obtained by punching the ear and then weighed. The inflammation induced by xylol was assessed by the change in the weight of ear punch of treated groups over control and this is called the edema index.

### Cotton pellet-induced granuloma in rats^10^:

Two sterilized cotton pellets, each weighing 10mg were implanted subcutaneously into axilla in anaesthetized rats. After treatment with test compounds, standard and vehicle for 10 days the rats were sacrificed. They were dissected to take out granuloma tissue and dried at 60° overnight to determine the dry weight. Results were expressed as mg/100 g.

### Writhing test^11^:

Acetic acid-induced writhing model was employed to evaluate the analgesic activity. The test compounds, standard and vehicle were administered orally to the mice and 30 min later 0.6% acetic acid solution (10 ml/kg.) was injected intraperitoneally. Nimesulide (50 mg/kg) was used as standard. The number of writhes induced in each mouse was observed for 10 min period starting 10 min after injection of acetic acid. The analgesic activity was expressed in terms of percentage inhibition of writhes produced by acetic acid.

### Rat caudal immersion method^12^:

The test compounds, standard and vehicle as given above were administered orally. The reaction time for withdrawal of tail was recorded after 60 min from the administration of test compounds. It was determined by immersing the tail up to the caudal portion (5 cm from the tip) in hot water (55±0.5°) and by noting the time taken to withdraw the tail clearly out of water. Observations were made at an interval of 30, 60, and 180 min after the initial reading.

### Antipyretic activity:

Yeast induced pyrexia was used to evaluate the antipyretic activity of the test compounds. The body temperature of each rat was recorded by measuring the rectal temperature at predetermined time intervals. Fever was induced by injecting 15% suspension of Brewer's yeast (*Saccharomyces cerevisiae*) following a standard method^12^. The rats were allowed to remain quiet in the cage for sometime. A thermister probe was inserted 3-4 cm deep into the rectum after fastening the tail to record the basal rectal temperature. The animals were then given a subcutaneous injection of 10 ml/kg of 15%w/v Brewer's yeast suspended in 0.5% w/v CMC solution and the animals were returned to their housing cages. Nineteen hours after yeast injection, the rats were again restrained in individual cages to record their rectal temperature. Immediately the test compounds and standard were administered orally at their respective doses. Rectal temperature of all the rats was recorded at 19 h immediately before the administration of test compounds, vehicle and paracetamol (50 mg/kg.) and again at 1hour intervals upto 3hours after the administration.

### Cyclooxygenase inhibition activity:

The colorimetric COX (ovine) Inhibitor Screening Assay utilizes the peroxidase component of cyclooxygenase. The peroxidase activity is assayed colorimetrically by monitoring the appearance of oxidized N,N,N,N-Tetramethyl-p-phenylenediamine (TMPD) at 590 nm. The estimation of COX-1 and COX-2 enzyme inhibitor activity was done using the kit supplied by Cayman Chemical (USA). The kit contained Assay buffer (10X), Heme, COX-1(Ovine), COX-2(Ovine), Arachidonic acid, Potassium hydroxide, Colorimetric substrate, 96 well plate.

### Statistical Analysis:

The Statistical analysis was performed by using One Way ANOVA followed by Dunnet's Test. and the P< 0.01 was taken as significant.

## RESULTS AND DISCUSSION

The effect of thiazolidin-4-one derivatives on carrageenan-induced paw edema and cotton pellet-induced granuloma in rats are mentioned in Table 2 and 3. All the eleven compounds B_1_ to B_11_ showed significant inhibition of edema and granuloma dry weight at both the doses tested (50 mg/kg and 100 mg/kg) in a dose-dependant manner. The maximum inhibition was observed at 3^rd^ h. The maximum inhibition of edema and reduction in dry weight was produced by compounds B_1_, B_3_, B_4_, B_6_, B_8_, B_10_, and B_11_ as compared to standard nimesulide. The results of effect of thiazolidin-4-one derivatives on xylol-induced ear edema in mice are given in Table 4 and analgesic activity of thiazolidin-4-ones in acetic acid induced writhing in mice and rat caudal immersion are mentioned in Table 5. All the test compounds also showed significant inhibition of edema in xylol induced mouse ear edema; however, it was less as compared to standard nimesulide. In acetic acid-induced writhing all the test compounds showed significant analgesic activity equal to standard nimesulide, however, in caudal immersion test none of the compounds showed any analgesic activity. In yeast induced pyrexia test compounds B_1_, B_2_, B_5_, B_6_, B_7_, B_8_ and B_11_ showed significant inhibition of pyrexia at higher dose of 100 mg/kg as compared to B_3_, B_4_, B_9_ and B_10_ (Table 6). In *in vitro* COX-1 and COX-2 enzyme inhibition assay, the test compounds B_1_, B_2_, B_5_, B_6_ and B_8_ showed maximum inhibition of COX-2 enzyme activity than the other test compounds and comparable to nimesulide. However, all the test compounds and standard did not inhibit the COX-1 enzyme activity (Table 7).

**TABLE 2:** EFFECT OF THIAZOLIDINONE DERIVATIVES ON CARRAGEENAN-INDUCED PAW EDEMA

Compound code	Dose (mg/kg)	Mean paw volume at different time intervals (ml)		Percentage inhibition of edema volume (ml)	
			
		1^st^ h	3^rd^ h	1^st^ h %	3^rd^ h %
Control	50	5.85±0.044	6.82±0.018	0.0	0.0
Nimesulide	50	4.97±0.014**	3.84±0.012**	15.1	43.7
B_1_	50	4.68±0.046**	5.46±0.064**	20.0	20.0
	100	4.72±0.064**	4.71±0.056**	19.4	31.0
B_2_	50	5.12±0.079**	4.02±0.040**	12.5	41.1
	100	4.74±0.027**	4.06±0.054**	18.9	40.5
B_3_	50	5.17±0.067**	4.34±0.061**	11.7	36.4
	100	5.09±0.039**	3.92±0.025**	13.0	42.6
B_4_	50	4.89±0.033**	4.19±0.061**	16.4	38.6
	100	4.73±0.024**	4.23±0.021**	19.1	37.9
B_5_	50	5.04±0.048**	4.39±0.037**	13.9	35.6
	100	4.90±0.039**	4.08±0.056**	16.2	40.2
B_6_	50	5.09±0.039**	4.29±0.032**	12.9	37.0
	100	4.94±0.040**	4.04±0.037**	15.5	40.8
B_7_	50	4.97±0.056**	4.29±0.032**	15.0	37.0
	100	4.88±0.030**	4.04±0.040**	16.6	40.8
B_8_	50	4.74±0.045**	4.56±0.033**	19.0	33.2
	100	4.90±0.030**	3.61±0.072**	16.3	47.1
B_9_	50	5.36±0.108**	4.21±0.031**	08.4	38.3
	100	4.63±0.045**	4.08±0.015**	20.9	40.2
B_10_	50	5.16±0.048**	4.77±0.052**	11.8	30.1
	100	5.02±0.091**	4.50±0.049**	14.2	34.1
B_11_	50	5.24±0.108**	4.46±0.091**	10.5	34.7
	100	5.02±0.027**	4.27±0.031**	14.2	37.4

N=6, Values are Mean±SEM

** *P*<0.01(significant), values are compared with control group

**TABLE 3:** EFFECT OF THIAZOLIDINONES ON COTTON PELLET INDUCED GRANULOMA IN RATS

Compound code	Dose (mg/kg)	Dry granuloma tissue weight (mg)	Percentage inhibition
Control	50	45.70±1.133	0.0
Nimesulide	50	25.82±1.398**	43.6
B_1_	50	29.53±1.062**	35.4
	100	27.56±1.133**	39.7
B_2_	50	30.18±1.573**	34.0
	100	28.61±2.461**	32.8
B_3_	50	30.74±1.141**	37.4
	100	28.00±1.716**	38.8
B_4_	50	29.68±2.008**	35.1
	100	26.30±1.322**	42.5
B_5_	50	31.51±1.171**	31.1
	100	28.58±0.9896**	37.5
B_6_	50	29.67±2.626**	35.1
	100	26.72±1.435**	41.5
B_7_	50	32.84±1.644**	28.2
	100	29.68±1.071**	35.1
B_8_	50	29.98±1.583**	34.4
	100	27.72±0.9156**	39.4
B_9_	50	29.85±1.612**	34.7
	100	28.76±0.6516**	37.1
B_10_	50	31.46±2.302**	31.2
	100	27.88±1.261**	39.0
B_11_	50	32.19±1.249**	29.6
	100	27.86±1.035**	39.1

N=6, Values are Mean±SEM

** *P*<0.01 (significant), values are compared with control group

**TABLE 4:** EFFECT OF THIAZOLIDINONE DERIVATIVES ON XYLOL-INDUCED EAR EDEMA IN MICE

Compound code	Dose (mg/kg)	Weight of the ear (mg)	Percentage inhibition
Control	50	8.68±0.414	0.0
Nimesulide	50	2.82±0.188**	67.51
B_1_	50	7.06±0.294**	28.54
	100	4.96±0.335**	49.79
B_2_	50	7.12±0.343**	27.93
	100	4.94±0.329**	50.00
B_3_	50	6.88±0.333**	30.36
	100	4.14±0.136**	58.09
B_4_	50	6.98±0.427**	29.35
	100	4.32±0.399**	56.27
B_5_	50	6.98±0.379**	29.35
	100	4.30±0.270**	56.47
B_6_	50	7.34±0.405**	25.70
	100	4.84±0.294**	51.01
B_7_	50	6.56±0.411**	33.60
	100	4.24±0.188**	57.08
B_8_	50	6.40±0.148**	35.22
	100	4.42±0.229**	55.26
B_9_	50	6.08±0.352**	29.95
	100	3.98±0.086**	54.14
B_10_	50	6.02±0.182**	30.64
	100	3.40±0.148**	60.89
B_11_	50	6.38±0.407**	26.49
	100	4.24±0.266**	51.15

N=6, Values are Mean±SEM

** *P*<0.01 (significant), values are compared with control group

**TABLE 5:** ANALGESIC ACTIVITY OF THIAZOLIDINONES IN ACETIC ACID-INDUCED WRITHING IN MICE AND RAT CAUDAL IMMERSION

Compound code	Dose (mg/kg)	Writhing response	Percentage inhibition	Caudal immersion reaction time (In sec.)
Control	50	36.00±0.70	0.0	1.05±0.022
Nimesulide	50	14.50±0.64**	59.8	4.05±0.010
B_1_	50	22.50±0.86**	37.5	1.90±0.02
	100	20.00±1.58**	44.5	1.85±0.08
B_2_	50	20.00±1.87**	44.5	1.70±0.04
	100	16.75±1.31**	53.5	1.72±0.02
B_3_	50	20.25±1.43**	43.8	1.60±0.05
	100	16.50±0.86**	54.2	1.70±0.06
B_4_	50	18.75±0.75**	48.0	2.02±0.04
	100	14.25±0.94**	60.5	2.08±0.02
B_5_	50	20.00±1.08**	44.5	1.90±0.12
	100	16.50±1.04**	54.2	1.80±0.14
B_6_	50	25.25±1.75**	29.9	2.10±0.08
	100	24.00±0.70**	33.4	2.14±0.06
B_7_	50	17.75±2.68**	50.7	2.14±0.06
	100	13.75±0.85**	61.9	1.98±0.02
B_8_	50	18.50±1.19**	48.7	1.80±0.12
	100	15.00±1.47**	58.4	2.06±0.18
B_9_	50	18.00±0.91**	50.0	1.52±0.06
	100	15.50±1.19**	57.0	1.40±0.02
B_10_	50	18.50±1.19**	48.7	1.62±0.06
	100	14.00±0.70**	61.2	1.52±0.04
B_11_	50	17.75±1.03**	50.7	1.42±0.02
	100	15.00±0.70**	58.4	1.38±0.04

N=6, Values are Mean±SEM

** *P*<0.01 (significant), values are compared with control group

**TABLE 6:** EFFECT OF THIAZOLIDINONE DERIVATIVES ON YEAST-INDUCED PYREXIA

Compound code	Dose (mg/kg)	Yeast induced Pyrexia (Temp. in °C)			
		
		0 h	1/2 h	1st h	3rd h
Control	50	37.66±0.26	37.48±0.22	37.24±0.15	36.66±0.18
Nimesulide	50	37.46±0.12	36.94±0.06*	36.66±0.07*	35.44±0.11**
B_1_	50	36.96±0.08	36.68±0.09**	36.42±0.08**	36.02±0.04**
	100	37.00±0.13	36.72±0.06**	36.32±0.05**	35.60±0.04**
B_2_	50	37.10±0.12	36.80±0.13*	36.60±0.10**	36.44±0.06
	100	37.08±0.10	36.72±0.08**	36.48±0.08**	35.46±0.14**
B_3_	50	37.26±0.16	36.90±0.14*	36.60±0.20**	36.32±0.12
	100	36.96±0.08	36.66±0.06**	36.22±0.06**	35.64±0.05**
B_4_	50	37.18±0.15	36.98±0.16	36.64±0.07*	36.28±0.04
	100	37.32±0.16	36.98±0.11*	36.62±0.05**	35.88±0.09**
B_5_	50	37.06±0.16	36.78±0.11*	36.64±0.15*	36.34±0.06
	100	37.24±0.15	36.88±0.13**	36.64±0.12**	35.74±0.09**
B_6_	50	37.48±0.24	36.96±0.21	36.56±0.18**	36.22±0.10*
	100	37.18±0.11	36.72±0.15**	36.40±0.13**	35.60±0.11**
B_7_	50	37.22±0.18	36.76±0.08**	36.48±0.03**	36.18±0.06**
	100	37.02±0.08	36.46±0.09	36.38±0.06**	35.56±0.08**
B_8_	50	37.18±0.18	36.80±0.10*	36.42±0.11**	36.20±0.06*
	100	37.14±0.12	36.72±0.05**	36.30±0.10**	35.56±0.12**
B_9_	50	37.14±0.12	36.82±0.09**	36.52±0.12**	36.10±0.08**
	100	37.24±0.11	36.86±0.08*	36.46±0.13**	35.78±0.18**
B_10_	50	37.64±0.08	37.04±0.06	36.54±0.06**	36.10±0.06*
	100	36.94±0.06	36.62±0.11**	36.26±0.10**	35.44±0.07**
B_11_	50	37.32±0.12	36.82±0.07**	36.58±0.03**	35.98±0.11*
	100	37.10±0.10	36.62±0.15**	36.20±0.07**	35.58±0.09**

N=6, Values are Mean±SEM

** *P*<0.01(significant)

* *P*<0.05(significant) values are compared with control group

**TABLE 7:** EFFECT OF THIAZOLIDINONE DERIVATIVES ON COX-1 AND COX-2 ENZYME INHIBITORY ACTIVITY

Compound code	Drug in μg/ml	Percentage Inhibition*	
		
		COX-1	COX-2
Background wells	-	-	-
100% initial activity wells	-	-	-
B_1_	10	08.176	44.391
B_2_	10	13.522	49.880
B_3_	10	12.839	41.050
B_4_	10	07.232	42.959
B_5_	10	01.759	46.778
B_6_	10	01.194	44.391
B_7_	10	02.974	38.663
B_8_	10	05.660	43.198
B_9_	10	01.542	11.697
B_10_	10	02.349	09.983
B_11_	10	02.843	15.295

* Average of 3 readings

**Fig. 1:**
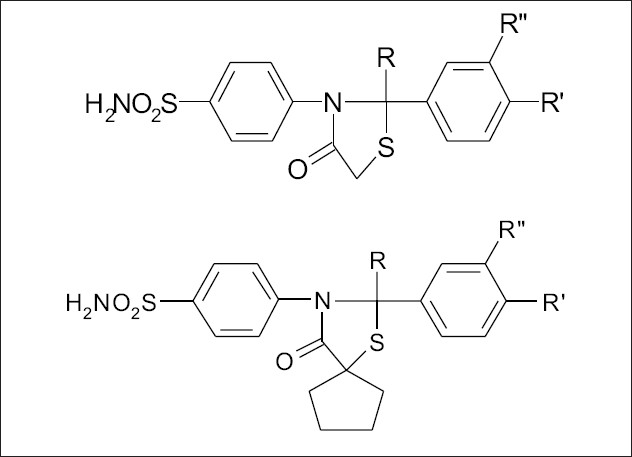
General structure of thiazolidinone derivatives.

In the present study, eleven derivatives of thiazolidine-4-ones synthesized (B_1_ to B_11_ ) from sulphanilamide, a well known antibacterial agent were evaluated for antiinflammatory, analgesic and antipyretic activities using various models. In all these compounds the free amine group is replaced by thiazolidine-4-one with functional group substitution at R, R_1_ and R_2_ positions (fig. 1). All the test compounds showed significant antiinflammatory, analgesic, antipyretic and COX-2 enzyme inhibition activity very much similar to nimesulide. However, substitution of functional groups at R-H, R_1_-OCH_3_ and R_2_-H in B_2_, R-H, R_1_-OCH_3_ and R_2_-OH in B_5_, R-H, R_1_-Cl and R_2_-H in B_6_, R-CH_3_, R_1_-NO_2_ and R_2_-H in B_7_, R-H, R_1_-F and R_2_-H in B_8_ showed significantly higher activity as compared to B_1_ where there is no substitution at both R and R_2_ positions. However, compounds B_3_ and B_4_ even though had substitution R-CH_3_, R_1_-Cl and R_2_-H in B_3_ and R-CH_3_, R_1_-OCH_3_ and R_2_-H in B_4_, did not show higher activity. Similarly in the spiro substituted compounds the substitution of functional groups R-CH_3_, R_1_-OCH_3_ and R_2_-H in B_11_ lead to an increased activity as compared to non-substituted B_9_ and B_10_ compounds_._ In general all the spiro substituted derivatives showed less activity, which may be attributed to the introduction of spiro group in thiazolidine-4-one that may interfere in the binding of test compounds to the enzyme. This shows that any substitution at the 5-position in the thiazolidine-4-one leads to decrease in activity, that may be because of the stearic hindrance caused by the substitution.

There is tremendous amount of experimental^13^ and theoretical^14^ works, focused on the study of COX-2 and the existence of high-resolution structural information on the binding site of NSAIDs, several aspects of binding mechanism of DUP-697 and related compounds to COX-2 remains unclear. Observing the data from literature^1^–^3^ reveals that empirical rules formulated for the given set of drugs are not agreeable when applied to a different set, even when both set of compounds share a common background. This suggests that subtle structural changes in binding site of COX-2 might occur to adopt its structure to the inhibitor. This might also be the reason for many diverse group of compounds reported to have antiinflammatory activity^4^–^7^. The marketed antiinflammatory drugs contain 2-phenyl rings attached to heterocyclic ring systems like thiophene, oxazolidinone, 1,2-pyrazole and 1,3 pyrazoles with a sulfonyl group and such a combination appears to be essential for specific COX-2 activity^3^,^14^. However, such drugs are also associated with severe adverse effects.

Therefore, from earlier reports and also from our results it can be substantiated that the COX-2 binding site may not be a rigid structure and might adopt to various related groups which may be the reason for thiazolidin-4-one derivatives tested in the present study for having action similar to nimesulide. However substitution at 5-position with spiro moiety resulted in reduced COX-1 and COX-2 inhibitory activity.

In conclusion several derivatives tested in this study showed maximum inhibition of COX-2 activity without inhibiting the COX-1 activity and results are comparable with that of nimesulide. The substitution at particular place i.e., R, R_1_ and R_2_ with the functional groups Cl, OCH_3_, NO_2_ and OH in the aromatic ring resulted in increased activity as compared to unsubstituted thiazolidin-4-ones and substitution at 5-position with spiro group did not improve the activity.
